# When You Hear Hoofbeats, It May Still Be the Medication: A Case of Aripiprazole‐Induced Hypersexuality Masquerading as Psychiatric Decompensation

**DOI:** 10.1155/crps/2595434

**Published:** 2026-10-25

**Authors:** Nana Cudjoe, Terrance C. Jenkins, Brittany Stallworth, Cansery Kathomi Mwai, Alainia Morgan

**Affiliations:** ^1^ Department of Psychiatry and Behavioral Sciences Morehouse School of Medicine Atlanta Georgia USA msm.edu; ^2^ Behavioral Health Clinic Grady Memorial Hospital Atlanta Georgia USA gradymem.org

## Abstract

Aripiprazole has a known association with impulse‐control disorders (ICDs), yet these adverse effects are frequently underrecognized and misattributed to psychiatric decompensation, particularly in women. We report a case of a 27‐year‐old African American woman with schizoaffective disorder who developed marked, new‐onset hypersexual behavior temporally associated with aripiprazole. Aripiprazole accounts for a majority of ICDs reported among this class of antipsychotics, likely due to its D2/D3 partial agonism. This case highlights the critical need for clinicians to maintain a high index of suspicion for medication‐induced ICDs when patients develop new impulsive or compulsive behaviors.

## 1. Background

Aripiprazole, a dopamine D2 partial agonist, has been associated with impulse‐control disorders (ICDs), including hypersexuality. Although recognized, these adverse effects are often underappreciated in acute settings and may be misattributed to primary psychiatric illness, leading to delays in appropriate management. Additionally, there is limited literature examining these presentations in African American women, where such behaviors may be underrecognized or misinterpreted, contributing to potential delays in diagnosis and treatment.

## 2. Case Presentation

We present the case of a 27‐year‐old African American woman with schizoaffective disorder, mixed type, who was hospitalized for worsening paranoid delusions and suicidal ideation. She had three prior psychiatric hospitalizations for paranoid delusions and decompensated psychosis with suicidal ideation. However, she had no documented or reported history of hypersexuality, compulsive sexual behavior, pathological gambling, or other impulse‐control behaviors.

The development of sexual behaviors represented a marked departure from her documented baseline. She had received paliperidone palmitate 234 mg intramuscularly every 28 days for several months. While receiving this regimen, she was generally adherent and functionally stable with mild residual perceptual disturbances but ongoing engagement in community‐based treatment. A recurrent pattern of worsening delusions and psychotic symptoms in the week before each scheduled injection was documented. Approximately 3.5 weeks before admission, she reported galactorrhea and urinary incontinence to her outpatient providers. Adjunctive oral risperidone 1 mg nightly was discontinued, and serum prolactin testing was ordered. Approximately 2.5 weeks before the admission, the prolactin concentration was reported as elevated at 117.20 ng/mL.

To address hyperprolactinemia, aripiprazole 10 mg orally once daily was added ~2 weeks before the admission while paliperidone palmitate treatment was ongoing. However, she reported inconsistent adherence to aripiprazole prior to admission. Her final paliperidone palmitate injection was administered ~10 days before the admission, with no subsequent injections because of intolerable symptoms associated with hyperprolactinemia. Aripiprazole 10 mg orally once daily was continued on admission and increased to 15 mg on hospital day (HD) 4, then to 20 mg on HD 7. On HD 8, the patient developed newly documented compulsive hypersexual behavior on the inpatient psychiatric unit. Clinical staff observed multiple episodes of daily compulsive masturbation, with individual episodes lasting several hours. The patient described the behavior as new and difficult to control. During clinical interviews, she was unable to be redirected from the behavior. She did not endorse any direct relationship between the behavior and psychotic content, and the repetitive, prolonged, and newly emergent compulsive sexual behavior was clinically distinct from her previously documented illness presentation.

Although her history of schizoaffective disorder, mixed type, required consideration of a mood‐related contribution to the behavioral change, the clinical documentation did not demonstrate a sustained syndromic manic presentation during the period when compulsive hypersexual behavior was most prominent. Specifically, there was no decreased need for sleep, increased energy, pressured speech, grandiosity, increased goal‐directed activity, or sustained psychomotor activation during this period. There was also no evidence of acute substance intoxication or withdrawal contributing to the presentation. During the admission, she exhibited paranoid and persecutory delusions and command auditory hallucinations. However, there was no identified sexual theme in the hallucinations or delusions to link the compulsive sexual behavior to psychotic symptoms. Divalproex sodium 1000 mg was initiated on HD 11 for impulsivity; however, compulsive hypersexual behaviors continued to be documented. Given the concern for a possible aripiprazole‐associated impulse control adverse effect, aripiprazole was reduced from 20 to 10 mg daily on HD 15 and discontinued on HD 18. Resolution of hypersexual behavior was documented on HD 20, 2 days after aripiprazole discontinuation [Table 1]. A subsequent trial of lurasidone was initiated and titrated to 120 mg; this resulted in worsening psychotic symptoms but no recurrence of hypersexual or impulse‐control behaviors. She was later cross‐titrated from lurasidone and divalproex to clozapine with improvement in psychosis. At the time of discharge, she demonstrated a return to baseline functioning with improved insight, and no recurrence of hypersexual behaviors was noted following the initial resolution.

**Table 1:** Clinical and medication timeline.

Time	Medication/clinical event	Hypersexual behavior	Clinical interpretation/outcome
Several months before admission	Paliperidone palmitate 234 mg IM every 28 days; generally adherent and functionally stable, with recurrent worsening of psychosis during the week before injections	No documented or reported hypersexuality or other impulse‐control behavior	Established pre‐aripiprazole behavioral baseline
~3.5 weeks before admission	Galactorrhea and urinary incontinence reported; adjunctive risperidone 1 mg nightly discontinued; prolactin ordered	None documented	Adverse effects prompted medication reassessment
~2.5 weeks before admission	Prolactin 117.20 ng/mL	None documented	Hyperprolactinemia confirmed
~2 weeks before admission	Aripiprazole 10 mg PO daily added while paliperidone exposure continued; preadmission adherence was inconsistent	None documented	Aripiprazole introduced before onset of new behavior
~10 days before admission	Final paliperidone palmitate 234 mg injection; no further injections because of hyperprolactinemia‐related symptoms	None documented	Residual long‐acting paliperidone exposure remained during early admission
HD 1	Aripiprazole 10 mg PO daily continued	No hypersexual behavior documented at admission	Inpatient administration established
HD 4	Aripiprazole increased to 15 mg daily	None documented	Dose escalation
HD 7	Aripiprazole increased to 20 mg daily	None documented before dose increase	Further dose escalation
HD 8	Aripiprazole 20 mg daily continued	New‐onset compulsive hypersexual behavior: multiple daily masturbation episodes, some lasting several hours; difficult to redirect	Marked departure from baseline, beginning 1 day after increase to 20 mg
HD 11	Divalproex sodium 1000 mg initiated for impulsivity	Compulsive hypersexual behavior persisted	Differentials included mood/psychotic symptoms versus medication‐associated impulse‐control disorder
HD 15	Aripiprazole reduced from 20 to 10 mg daily	Behavior continued to be monitored	Dose reduction due to concern for aripiprazole‐associated impulse‐control adverse effect
HD 18	Aripiprazole discontinued	Hypersexual behavior remained the target suspected adverse effect	Medication dechallenge
HD 20	Aripiprazole remained discontinued	Resolution of hypersexual behavior documented	Resolution 2 days after discontinuation supported a medication‐associated adverse effect
Subsequent inpatient course	Lurasidone initiated and titrated to 120 mg; later cross‐titrated from lurasidone and divalproex to clozapine	No recurrence of hypersexual or other impulse‐control behavior	Psychosis worsened on lurasidone without recurrence of hypersexuality, helping distinguish the behavior from psychotic decompensation
Discharge	Clozapine treatment associated with improvement in psychosis	No recurrence after initial resolution	Return toward baseline functioning with improved insight

## 3. Discussion

According to the Food and Drug Administration‐approved drug label, aripiprazole carries a specific warning regarding pathological gambling and other impulsive or compulsive behaviors, including compulsive sexual urges [1]. These effects are primarily attributed to its partial agonism at the dopaminergic D2/D3 receptors, although 5‐HT1A receptor agonism is also emerging as a plausible mechanism contributing to impulsivity with antipsychotics. While several other dopamine receptor partial agonist antipsychotics such as brexpiprazole and cariprazine share similar receptor affinities, a recent study suggested that aripiprazole accounted for ~94% of antipsychotic‐associated ICDs in this class [2]. Sexual dysfunction associated with antipsychotics is often underrecognized in women, despite multiple studies demonstrating strong association with aripiprazole and compulsive sexual behaviors, particularly in women aged 19–41 years of age [3]. In African American women, medication‐induced hypersexual behaviors may not only be underrecognized but also be underreported. These behaviors may be misattributed to sociocultural stereotypes, baseline personality characteristics, or trauma‐related factors, contributing to delayed identification and potential bias in clinical interpretation.

Gambling is the most noted ICD associated with aripiprazole, followed by hypersexuality. Symptoms have frequently been reported to emerge within ~30 days (about 4 and a half weeks) of initiation or following dose increases and can occur in patients with or without a prior history of impulsive behaviors [4]. When this adverse effect is suspected, dose reduction should first be attempted, as some cases may resolve with lower doses [1]. Persistent or severe symptoms warrant discontinuation of aripiprazole, with symptom resolution typically occurring within days to weeks [5]. Many patients may experience this adverse effect in the outpatient setting and may not recognize these behaviors as medication‐related. Therefore, it is important for clinicians not only to be cognizant of this potential effect but also to routinely educate patients and their caregivers to be vigilant of new impulsive behaviors.

## 4. Conclusion

This case highlights the potential for aripiprazole to induce severe impulse‐control disturbances, including hypersexuality. Importantly, such presentations may be misinterpreted as psychiatric decompensation, mania, or behavioral disturbance intrinsic to the underlying illness. In this patient, the temporal association with aripiprazole exposure, the absence of prior history of impulse‐control behaviors, and resolution after discontinuation support a medication‐induced etiology. A Naranjo Adverse Drug Reaction Probability Scale score of 8 indicated a probable adverse drug reaction. These findings underscore the importance of recognizing medication‐induced etiologies and prioritizing the discontinuation of the offending agent. This case also illustrates the clinical challenges that arise when system‐level or diagnostic anchoring delays appropriate intervention. Clinicians should maintain a high index of suspicion for medication‐induced ICDs in patients treated with aripiprazole, particularly when new‐onset hypersexual behaviors emerge. When “hoofbeats” are heard, common explanations such as illness relapse should be considered, but medication effects must not be overlooked.

## Funding

No funding was received for this study.

## Disclosure

The authors have no financial or other relationships to disclose.

## Ethics Statement

This case report was prepared in accordance with applicable ethical standards for the reporting of individual patient cases. All identifying information has been removed or sufficiently deidentified to protect the patient’s privacy and confidentiality. Institutional review board (IRB) review was not required under applicable institutional policies for publication of this deidentified case report.

## Consent

Informed consent was received from the patient to publish this case report, and the information has been deidentified to protect the patient’s anonymity.

## Conflicts of Interest

The authors declare no conflicts of interest.

## Data Availability

Data sharing is not applicable to this article, as no datasets were generated or analyzed during the current study.
